# Particulate Matter Emissions of Turkish Cigarettes in Comparison to German Brands – An Aerosol Spectrometric Study

**DOI:** 10.1177/1179173X261473110

**Published:** 2026-09-08

**Authors:** Markus Braun, Nicole Merkel, Doris Klingelhöfer, Janis Dröge, David A. Groneberg

**Affiliations:** 1 Institute of Occupational Medicine, Social Medicine and Environmental Medicine, 9173 Goethe University Frankfurt, Frankfurt am Main, Germany

**Keywords:** environmental tobacco smoke, passive smoke, fine particles, tobacco control, indoor air, Turkish tobacco farming, oriental tobacco

## Abstract

**Background:**

Türkiye has one of the highest smoking prevalence rates among OECD countries, leading to quite increased exposure to secondhand smoke (SHS), which significantly contributes to indoor particulate matter (PM) that has substantial adverse effects on health. As a nation with a long-standing tradition of tobacco cultivation and consumption, Türkiye has historically struggled to implement effective tobacco control strategies.

**Methods:**

In a closed 2.88 m^3^ measuring chamber, tobacco smoke from 11 Turkish cigarette brands and, for comparison, four German equal brands, and the 3R4F reference cigarette was produced. PM_10_, PM_2.5_, and PM_1_ were measured using laser aerosol spectroscopy. Pearson correlations assessed links between PM levels and tobacco weight, packing density, and filter and tobacco rod lengths.

**Results:**

Concentrations of PM varied from 1044 to 1677 μg/m^3^ for PM_10_ and from 1043 to 1675 μg/m^3^ for PM_2.5_, with most emissions originating from the detrimental PM_1_ fraction, ranging from 994 to 1548 μg/m^3^. Five Turkish brands exhibited the highest emissions of PM_10_ and PM_2.5_, four brands concerning PM_1_, respectively, surpassing the levels emitted by the reference cigarette. All four German brands produced lower PM. The study couldn’t report correlations between measured mean PM concentrations and measured tobacco weights, calculated filling densities, or measured filter and tobacco rod lengths.

**Conclusion:**

PM measured for all tested cigarette brands was significantly high, particularly for the quite harmful PM_1_ fraction. Smoking in cars, similar in size to the test chamber, puts passengers, often children, at risk. A global ban on smoking in vehicles would help protect them. While public smoking is often restricted, unregulated home smoking still exposes people to dangerous PM levels. The study findings highlight the urgent need for robust tobacco control policies in Türkiye and globally, as well as a heightened public awareness of indoor PM pollution resulting from smoking.

## Introduction

Türkiye has a long history of smoking and the cultivation of tobacco beginning in the 1570s.^1^ The Ottoman Empire sought to control tobacco production and trade. Tobacco production and processing were regulated and overseen by the state until the establishment of the Tobacco Monopoly (Tütün Tekeli) in 1862. In 1884, the French-founded Reji Company then took over and provided a revenue share to the empire, establishing tobacco factories that produced highly sought-after Turkish tobacco in Europe.^2,3^ After the establishment of the Turkish Republic, the Reji company was compensated, and its rights and obligations were transferred to government control in 1925. By 1926, the State Monopoly (TEKEL) began overseeing tobacco production, cigarette manufacturing, and the pricing and sale of tobacco products.^2^ In 1979, the government reformed its tobacco farming, production, and sales policies to permit domestic and international tobacco production and distribution. Then, it allowed international companies to import their products in 1984, establish their own distribution networks in 1986, and set up production facilities in 1991. As a result, tobacco imports increased, and Virginia and Burley tobacco began to be cultivated instead of hitherto dominating Oriental tobacco.^2^ Türkiye’s tobacco sector had undergone a market-oriented transformation due to neoliberal policies.^4^ State control over tobacco production and cigarette manufacturing had gradually diminished, allowing transnational companies to take charge.^5^ British American Tobacco (BAT) purchased TEKEL in 2008.^6^ This shift has increased cigarette consumption. According to the Türkiye Health Survey (THS), the smoking prevalence of Turkish daily smokers 15 years of age and older was 28.3% in 2022, with 41.3% for males and 15.5% for females.^7^ The 2016 Global Adult Tobacco Survey (GATS) yielded similar results: 31.6% overall daily smokers, with 44.1% of men and 19.2% of women.^8^ This puts Türkiye in third place among OECD countries in terms of tobacco consumption, with figures significantly exceeding the OECD average of 16%.^9,10^ Meanwhile, demand-side policies to reduce consumption, implemented since the mid-1990s, have had limited effectiveness amid rising production and trade.^5^ Türkiye is still a tobacco-producing country today. Data from 2023 indicates that the global market share of tobacco production is approximately 1.4%.^11^ Currently, Türkiye is globally the leading country in the cultivation of Oriental tobacco.^12^

Türkiye joined the Framework Convention on Tobacco Control (FCTC) of the World Health Organization (WHO) in 2005. By July 1, 2015, it was the only country to implement all six MPOWER ‘best buy’ tobacco control measures. However, tobacco use remains highly prevalent in the country.^13,14^ Elbek et al (2014)^15^ stated that Turkish practice shows that smoking bans and restrictions exist only in law, with violations becoming the norm, while compliant units are exceptions. They continued that this stems from various government bodies, especially the Ministry of Interior, failing to fulfill legal obligations—unlike the Ministry of Health, and that the weak legal standing of Provincial Tobacco Control Committees, reliance on local administrative officials, and lack of support from law enforcement have rendered “Smokeless air field” initiatives ineffective. They demanded to enhance the accountability of all public authorities, not just the Ministry of Health.^15^ The ambition of the tobacco industry is to make money, while tobacco control efforts should focus on curbing this ambition. A key weakness in Türkiye’s tobacco control policies is that this circumstance was not primarily targeted.^15^

In contrast, Germany’s tobacco control measures have evolved more gradually and have been debated over a longer period of time. Despite having implemented comparatively weaker advertising restrictions for many years, Germany enjoys a lower smoking prevalence today (18.8% 15+ years old smokers in 2022, with 21.3% men and 16.4% women).^16-18^ While Türkiye has officially adopted a more thorough tobacco control package aligned with the FCTC than Germany, it continues to face higher rates of smoking and specific challenges, including narghile consumption and illicit trade of tobacco products.^9^

The health risks linked to air pollution, particularly particulate matter (PM), are well-documented, with PM contributing significantly to non-communicable diseases that rank among the leading causes of death.^19,20^ PM can also trigger neurological and psychiatric issues, such as autism, anxiety, and Attention Deficit Hyperactivity Disorder (ADHD).^21^ Additionally, exposure to PM during pregnancy poses health risks to infants.^22^ PM_1_ is particularly harmful as such small particles can deeply penetrate the respiratory system.^23^ In homes of smokers, smoking is the main source of PM.^24^ Prior studies have identified very high concentrations of PM, especially PM_1_, from tobacco smoking, with variations in emissions often related to the origin of tobacco products used.^25-28^

Considering the unique aspects of Turkish tobacco consumption and production, the issue of PM pollution resulting from smoking Turkish cigarettes becomes relevant. To our knowledge, this is the first study investigating PM emissions from Turkish tobacco products. This investigation, part of the Tobacco Smoke Particles and Indoor Air Quality (ToPIQ) studies,^25-30^ provides data on indoor PM levels from Turkish cigarettes and, for comparison, German cigarettes and a reference cigarette. This information is significant for both policymakers and the public. Understanding the levels of PM generated by smoking tobacco products, particularly indoors, can aid in enhancing Turkish as well as global tobacco control efforts.

## Method

### Tobacco Products

PM was measured in the smoke of 16 different filter-tipped cigarette brands, with 23 to 30 single cigarettes each brand. Eleven of these brands were sourced from Türkiye. For comparison, four German brands (counterparts to four Turkish brands with identical names) and the reference cigarette 3R4F from the Kentucky Tobacco Research and Development Center at the University of Kentucky, USA^31^ were also examined. Table 1 presents the origin and attributes of the cigarettes analyzed.

**Table 1.** Characteristics of the cigarettes tested

Brand (Manufacturer)	Country of origin	Length [mm]	Filter length [mm]	Diameter [mm]	Tobacco weight [mg]	Filling density [mg/cm^3^]
Reference Cigarette 3R4F (A)	USA	84	27	8	713.2	249
Tekel 2000 Mavi (B)	Türkiye	82	21.6	7.4	565	217
Muratti Rosso (C)	Türkiye	82.6	26.8	7.8	531.2	199
Monte Carlo Slender Red (D)	Türkiye	82	26.6	7	424	199
Kent Blue (B)	Türkiye	82.2	20.2	7.6	531.8	189
Viceroy Navy (B)	Türkiye	82.2	21.8	7.8	548.4	190
Lark Blue (C)	Türkiye	82	22	8	530.2	176
Parliament Night Blue (C)	Türkiye	82.6	21.8	7.8	526	181
L&M Red Label (C)	Türkiye	82.2	20.4	7.4	565.2	213
Camel Yellow (D)	Türkiye	82.2	20.8	7.6	656.6	236
Marlboro Red (C)	Türkiye	83	21	8	571.8	183
Davidoff Ivory (E)	Türkiye	92	25	7.6	648.6	213
L&M Red (C)	Germany	82.6	21	8	602.2	194
Camel Yellow (D)	Germany	82.4	21	7.8	570.6	194
Marlboro Red (C)	Germany	83	21	7.8	576.6	195
Davidoff Gold (E)	Germany	92	26.8	8	624.4	191

Manufacturer: A = Kentucky Tobacco Research and Development Center, University of Kentucky. B = British American Tobacco (BAT). C = Philip Morris International (PMI). D = Japan Tobacco International (JTI). E = Imperial Brands. Dimensions, weight, and filling density are the mean values of five randomized chosen cigarettes of each brand.

### Experimental Setup

The measurements were conducted in a sealed chamber with a volume of 2.88 m^3^, which housed the Automatic Environmental Tobacco Emitter (AETSE). The AETSE was operated from outside the chamber, ensuring that no individuals were exposed to tobacco smoke during the process. This programmable smoke pump was developed by Schimpf-Ing Trondheim (Norway)^32^ to simulate the act of smoking tobacco products. The AETSE consists of a microcontroller that specifies the smoking protocol and features a stepper motor capable of precisely moving a 200 ml glass syringe within a carriage. This syringe is linked to the mouthpiece of the tobacco product via a polyamide tube. Two valves regulate the airflow when the smoke pump pulls on the lit tobacco product, and when the mainstream smoke is expelled back into the chamber. The burning tobacco product produces side-stream smoke throughout the smoking process.

### Smoking Protocol

The smoking protocol is based on the ToPIQ methodology from former studies^25-30^ and includes three phases: combustion, post-combustion, and ventilation phases. Initially, the cigarettes to be analyzed were ignited. After two initial puffs, seven standard puffs were taken at a rate of two per minute. Each puff had a volume of 40 ml and lasted three seconds. The cigarettes were extinguished after four minutes. After an additional 6 minutes (at least), an industrial radial fan was used to ventilate the chamber, preparing the air for the subsequent measurement. This process ensured that there were 10 minutes of precise measurement data for each tobacco product, along with smoke-free cabin air for the following. The smoking protocol provided a standardized approach for measuring PM by smoking each tobacco product consistently. It based on established protocols from the International Organization for Standardization (ISO Standard for Machine Smoking of Cigarettes, which involves a 35 ml puff volume at a rate of 1 puff per minute)^33^ and the WHO’s standard operating procedure for intensive smoking of cigarettes (which uses a 55 ml puff volume at a rate of 2 puffs per minute),^34^ as well as insights gained from observing real smokers.^29^

### Laser Aerosol Spectrometer

The used Laser Aerosol Spectrometer (LAS) Model 1.109 from Grimm Aerosol Technik GmbH & Co KG (Ainring, Germany) can detect airborne particles ranging in size from 0.25 to 32 µm.^35^ It presents the data in terms of dust mass fractions according to the United States Environmental Protection Agency (US EPA), classifying particles into PM_10_ (aerodynamic diameters ≤ 10 µm), PM_2.5_ (≤2.5 µm),^36^ and PM_1_ (≤1 µm). Compressed air was used to dilute the sampled air 1:10 with the Grimm dilution system VKL mini (Model 7.951), ensuring that the LAS measuring cell was safeguarded from high particle concentrations. The suction point of the LAS was located approximately 38 cm from the cigarette and 42 cm from the outlet valve, both at the same height.

### Data Processing

The data output for PM_10_, PM_2.5_, and PM_1_ were set to 6-second intervals, resulting in a 10-minute data block for each cigarette containing 101 individual measurement values. Statistical analyses were performed using GraphPad Prism version 11 (GraphPad Software, La Jolla, California, USA). We calculated the mean concentrations (Cmean) of PM_10_, PM_2.5_, and PM_1_ for each individual cigarette tested, followed by outlier detection (ROUT and Grubbs tests) and assessment of normal distribution. The criteria for normal distribution using four statistical tests for normal distribution (D’Agostino & Pearson, Anderson-Darling, Shapiro-Wilk, and Kolmogorov-Smirnov) were not met for two cigarette brands (p < 0.05, supplement text file Table S1 for PM_10_). Consequently, the data from each brand were analyzed using the non-parametric Kruskal-Wallis test and the following post-hoc Dunn’s multiple comparisons test to conduct pairwise comparisons of the mean concentrations of all 16 cigarette brands among themselves. The Dunn’s test calculates the significance levels of the respective group pairs. For correlation analyses, normality tests (likewise D’Agostino & Pearson, Anderson-Darling, Shapiro-Wilk, and Kolmogorov-Smirnov) of the mean values of PM, tobacco weights, calculated filling densities, and measured filter and tobacco rod lengths of all 16 cigarette brands were passed. As a result, Pearson correlation analyses were carried out to assess the relationship between the C_mean_ of PM_10_, PM_2.5_, and PM_1_ and measured tobacco weights, calculated filling densities, and measured filter and tobacco rod lengths. A significance level of α = 0.05 was established for all statistical tests. Additionally, the percentages of PM_10-2.5_, PM_2.5-1_, and PM_1_ fractions were calculated.

## Results

### PM Mean Concentrations and Percentage Differences to the Reference Cigarette

**Figure 1.**
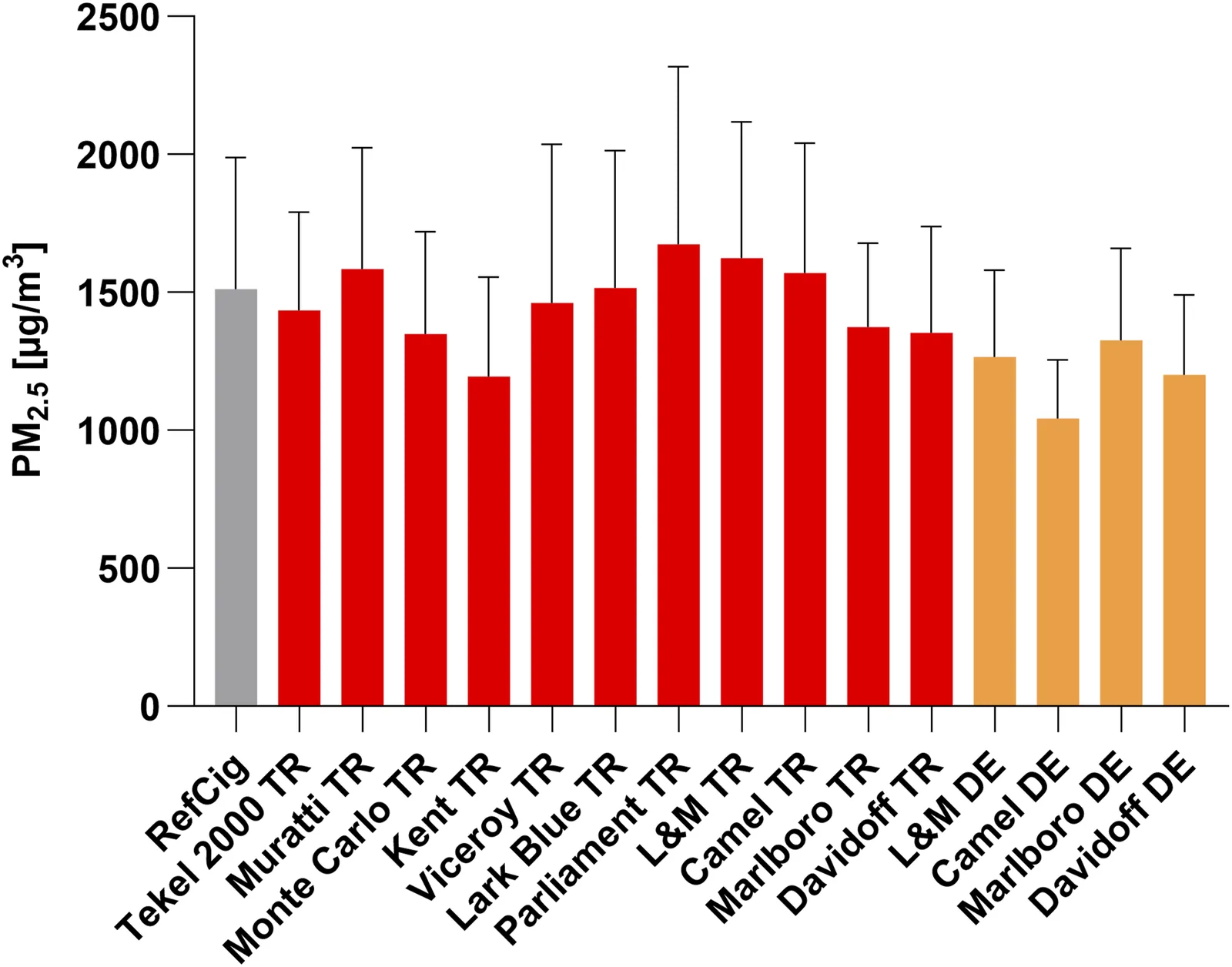
Bar graphs with marked ranges of Cmean PM2.5 of all cigarette brands tested. The red-colored bars refer to the Turkish brands, the orange-colored bars to the German brands. TR: Türkiye. DE: Germany

Table 2 presents the PM C_mean_ of all cigarette brands examined. Figure 1 illustrates the PM_2.5_ mean and maximum concentrations of all tested cigarette brands. The measured mean concentrations of PM ranged from 1044 µg/m^3^ to 1677 µg/m^3^ for PM_10_, 1043 µg/m^3^ to 1675 µg/m^3^ for PM_2.5_, and 994 µg/m^3^ to 1548 µg/m^3^ for PM_1_. The percentage differences compared to the reference cigarettes varied from -31% to +11% for PM_10_, from -31% to +11% for PM_2.5_, and from -31% to +7.5% for PM_1_. Five Turkish brands (concerning PM_10_ and PM_2.5_), respectively four (concerning PM_1_), emitted the most PM, meaning more PM than the reference cigarette, but without significance. Regarding PM_10_ and PM_2.5_, one Turkish and two German brands, in terms of PM_1_, one Turkish and three German brands, emitted significantly lower PM compared to the reference cigarette.

**Table 2.** Mean concentrations (C_mean_) for PM_10_, PM_2.5_, and PM_1_, along with standard deviation (SD) and the percentage distribution patterns of the particle fractions PM_1_, PM_2.5-1_, and PM_10-2.5_ across all tested cigarette brands

Brand (n), origin	PM_10_ [µg/m^3^]	PM_2.5_ [µg/m^3^]	PM_1_ [µg/m^3^]	PM_1_ [%]	PM_2.5-1_ [%]	PM_10-2.5_ [%]
Reference Cigarette 3R4F (30), USA	1512.3 ± 242.5	1510.9 ± 241.9	1439.0 ± 221.7	95.2	4.75	0.093
Tekel 2000 Mavi (25), Türkiye	1437.7 ± 161.9 (-4.9%, ns)	1435.4 ±161.4 (-5.0%, ns)	1341.2 ± 143.2 (-6.8%, ns)	93.3	6.55	0.160
Muratti Rosso (25), Türkiye	1586.9 ± 269.7 (+4.9%, ns)	1584.6 ± 268.9 (+4.9%, ns)	1482.9 ± 239.8 (+3.1%, ns)	93.4	6.41	0.145
Monte Carlo Slender Red (23), Türkiye	1350.5 ± 141.8 (-10.7%, ns)	1348.6 ± 141.5 (-10.7%, ns)	1261.0 ± 125.5 (-12.4%, ns)	93.4	6.49	0.141
Kent Blue (25), Türkiye	1195.4 ± 129.8 (-21.0%, ***)	1193.9 ± 129.4 (-21.0%, ***)	1134.2 ± 114.7 (-21.2%, ***)	94.9	4.99	0.125
Viceroy Navy (24), Türkiye	1463.4 ± 223.6 (-3.2%, ns)	1461.1 ± 223.3 (-3.3%, ns)	1381.2 ± 205.0 (-4.0%, ns)	94.4	5.46	0.157
Lark Blue (25), Türkiye	1517.3 ± 261.9 (+0.3%, ns)	1515.6 ± 261.5 (+0.3%, ns)	1422.8 ± 231.1 (-1.1%, ns)	93.8	6.12	0.112
Parliament Night Blue (25), Türkiye	1677.2 ± 257.7 (+10.9%, ns)	1674.6 ± 256.7 (+10.8%, ns)	1547.6 ± 220.6 (+7.5%, ns)	92.3	7.57	0.155
L&M Red Label (25), Türkiye	1626.2 ±187.3 (+7.5%, ns)	1624.1 ± 186.8 (+7.5%, ns)	1511.9 ± 160.1 (+5.1%, ns)	93.0	6.90	0.129
Camel Yellow (25), Türkiye	1571.3 ± 266.6 (+3.9%, ns)	1569.4 ± 266.0 (+3.9%, ns)	1471.2 ± 239.0 (+2.2%, ns)	93.6	6.25	0.121
Marlboro Red (25), Türkiye	1376.1 ± 139.7 (-9.0%, ns)	1374.3 ± 139.4 (-9.0%, ns)	1286.8 ± 121.1 (-10.6%, ns)	93.5	6.36	0.131
Davidoff Ivory (25), Türkiye	1354.6 ± 143.0 (-10.4%, ns)	1353.4 ± 143.0 (-10.4%, ns)	1291.0 ± 129.8 (-10.3%, ns)	95.3	4.61	0.089
L&M Red (25), Germany	1266.7 ± 199.7 (-16.2%, ns)	1265.0 ± 199.1 (-16.3%, ns)	1183.8 ± 170.0 (-17.7%, *)	93.5	6.41	0.134
Camel Yellow (25), Germany	1043.9 ± 135.8 (-31.0%, ***)	1043.2 ± 135.7 (-31.0%, ***)	993.7 ± 124.0 (-30.9%, ***)	95.2	4.74	0.067
Marlboro Red (25), Germany	1328.8 ± 189.2 (-12.1%, ns)	1327.0 ± 188.7 (-12.2%, ns)	1232.4 ± 164.9 (-14.4%, ns)	92.7	7.12	0.135
Davidoff Gold (25), Germany	1202.9 ± 137.0 (-20.5%, ***)	1201.7 ± 136.7 (-20.5%, ***)	1138.9 ± 119.1 (-20.9%, ***)	94.7	5.22	0.100

C_mean_ is rounded to one decimal place. N = number of cigarettes tested. The percentage differences in C_mean_ of the Turkish and German brands compared to the reference cigarette are provided in brackets, along with the corresponding significance levels (Dunn’s test). Significance levels: ns = not significant; * = significant (p = 0.01 to 0.05); *** = very significant (p < 0.001).

### Comparison of German and Turkish Cigarette Brands

When comparing the four German brands with their Turkish counterparts, all German brands emitted lower PM than the Turkish brands, two with high significance and two without. In percentages, these four Turkish brands emitted between 3.6% and 51% more PM_10_, between 3.6% and 50% more PM_2.5_, and between 4.4% and 48% more PM_1_ than their German counterparts.

### Particle Distribution

Table 2 presents the percentage distributions of the PM fractions PM_10-2.5_, PM_2.5-1_, and PM_1_. Most particles released by all brands belonged to the PM_1_ fraction, accounting for 92 to 95%. The percentages of the PM_2.5-1_ and PM_10-2.5_ categories were distinctly lower, ranging from 4.6 to 7.6% and less than 0.16%, respectively.

### Results of Correlation Analyses (Pearson)

No correlations between C_mean_ (PM_10_, PM_2.5_, and PM_1_) and the measured tobacco weights, calculated filling densities, or measured filter and tobacco rod lengths were found (p > 0.05).

## Discussion

In Türkiye, both active and passive smoking are extremely prevalent, leading to significant PM pollution from tobacco products, particularly indoors. This study provides strong evidence of this issue. Previous ToPIQ studies have already demonstrated that cigarettes release substantial amounts of PM.^25-30^ This is also applicable to all Turkish and German brands examined in this study. Notably, among the 16 cigarette brands tested, five Turkish brands produced the highest levels of PM. Among the five brands that emitted the least PM were all four German brands and one Turkish brand, with the four German brands emitting less PM than their comparable Turkish counterparts. In this respect, this study identified country-specific differences in PM emissions, consistent with two former ToPIQ studies.^25,27^ Such differences can be ascribed to factors like cigarette construction, including variations in tobacco blends, additives, and filter designs, as indicated by previous research.^37-40^ For instance, both studies and internal tobacco industry documents have shown that the presence of additives is associated with increased PM levels.^37,39,41^ Conversely, another study found no impact from additives, suggesting instead that the cigarette filter influences PM concentrations.^40^ Additionally, lower production standards among local manufacturers or the use of specific tobacco products, like clove cigarettes, may contribute to a higher PM burden from those products.^26^ The present investigation revealed no statistically significant correlations between PM emissions and the measured tobacco weights, computed filling densities, or measured filter and tobacco rod lengths across the analyzed samples. It should be noted, however, that the statistical power of the Pearson correlation analyses—conducted with a sample size of *N* = 16 (representing distinct cigarette brands)—remains relatively low. Consequently, further research is warranted, extending beyond the scope of Turkish-manufactured products to elucidate potential relationships through comprehensive assessments of manufacturing processes and tobacco blend compositions.

Like all prior ToPIQ studies, this analysis primarily aimed to compare the PM concentrations of the brands examined with one another and with a reference cigarette.^25-30,39,40^ Absolute PM data was provided for comparison with specific guidelines. For example, the 2021 WHO Global Air Quality Guidelines recommend that annual mean PM_10_ concentrations remain below 15 µg/m^3^ and PM_2.5_ below 5 µg/m^3^ (with short-term 24-hour mean PM_10_ concentrations below 45 µg/m^3^ and PM_2.5_ below 15 µg/m^3^).^19^ In this context, this study highlights the significant PM burden resulting from smoking, both for the smoker and those exposed to secondhand smoke (SHS). Most particles detected (>92%) fall within the PM_1_ fraction, as noted in earlier ToPIQ studies.^25-30,39,40^ These fine particles can penetrate deeply into the respiratory tract, making them particularly hazardous to the health of smokers as well as non-smokers who are exposed to SHS.^23^ Given that the measuring chamber used had a volume of 2.88 m^3^, comparable to that of a compact car,^42^ the health risks associated with smoking in vehicles are particularly alarming. The PM concentrations recorded inside a closed vehicle cabin with ventilation turned off were like those measured in the study’s measurement cabin.^43,44^ Additionally, another study indicated that smoking inside a car, especially with windows closed, resulted in elevated PM concentrations.^45^ A global ban on smoking in cars is long overdue, particularly considering that passengers are often children who require protection from SHS, PM, and the related health risks. Considerable efforts are necessary to devise targeted interventions to mitigate exposure to SHS in both homes and vehicles.^46^

Compared to road traffic, the vast PM burden caused by smoking in closed spaces is blatantly obvious. For example, a study in an urban city showed mean values of approximately 11, 14, and 24 µg/m^3^ PM_1_, PM_2.5_, and PM_10_, respectively.^47^ Another study presented, depending on the time of the day, mean concentrations of approximately 21, 36, and 84 µg/m^3^ PM_1_, PM_2.5_, and PM_10_, respectively, in urban areas, and 27, 39, and 74 µg/m^3^ PM_1_, PM_2.5_, and PM_10_, respectively, in suburban areas.^48^ By comparison, the respective PM mean values measured in this study were distinctly higher (up to 1548, 1675, and 1677 µg/m^3^ for PM_1_, PM_2.5_, and PM_10_, respectively) and are in line with previous ToPIQ studies.^25-30^ An observational study in a home of a real smoker reported an 1 h median of approximately 573 µg/m^3^ PM_2.5_, showing the vast burden of smoking in rooms.^49^ Naturally, the ambient background particle exposure is comparatively lower. For example, a study conducted in an inner-city area with no traffic found average PM levels of approximately 7, 10, and 20 µg/m^3^ PM_1_, PM_2.5_, and PM_10_, respectively.^47^

The results of this study emphasize the urgent need for regulations on tobacco use indoors and in vehicles, particularly when children and pregnant women are present. It is crucial to inform the public about the significant PM burden caused by smoking. Early education initiatives such as the Education Against Tobacco (EAT) prevention program could be effective in reducing smoking rates.^50^ There is a need for legislative action concerning occupational safety. The EU directive on pregnant workers currently, for example, does not list passive smoke among the substances that must be assessed or are prohibited for pregnant women in the workplace.^51^ Further studies have also highlighted the importance of tobacco tax policies, advocating for the implementation of high specific taxes to lower tobacco consumption.^52,53^

The experimental design of this study, utilizing the AETSE, differed from typical human smoking behavior, as real smokers were not involved due to ethical considerations. Individual smoking practices vary widely in terms of the number, frequency, and intensity of puffs taken, making such behaviors non-standardized. This variation may lead to discrepancies between the measured PM concentrations and actual exposure in real-world environments. However, standardization is crucial for allowing comparisons among different tobacco products. The modified smoke protocol employed in this study diverged from the protocols established by the International Organization for Standardization (ISO Standard for Machine Smoking of Cigarettes),^33^ the Health Canada Intense smoking regime,^54^ or the WHO standard operating procedure for intensive cigarette smoking,^34^ while also reflecting the behaviors of actual smokers.^29^ The indoor volume of the chamber used in this study (2.88 m^3^) more closely resembles real-life smoking scenarios, such as smoking in vehicles, compared to smoking machines. However, the chamber does not adequately replicate the complex dynamics of air exchange, ventilation, and environmental factors found in actual indoor settings. These differences from established smoking protocols or actual smoker behaviors may limit comparability. Nonetheless, it is important to note that a definitive “gold standard” for smoking protocols has yet to be established.^55^

Inhaled tobacco smoke (mainstream smoke) becomes humidified in the smoker’s respiratory tract, resulting in a hygroscopic increase in the size of exhaled particles by approximately 1.5 times, with some particles remaining in the respiratory system.^56,57^ SHS comprises only about 15% mainstream smoke, while the remaining 85% is side-stream smoke.^58^ Thus, the tobacco smoke generated by the AETSE closely resembles real SHS, without any individuals being exposed to tobacco smoke. Nonetheless, the particle count measured may be slightly elevated, with a tendency toward smaller particles compared to measurements taken from real smokers.

Another limitation of this study was the limited measurement range of the LAS, which spanned from 0.25 µm to 32 µm.^35^ Previous research has reported mean sizes or diameters of tobacco smoke particles falling within ranges such as 0.1 to 1 µm, 0.02 to 2 µm, or 0.1 to 0.5 µm.^59-62^ This suggests that most of the particles relevant to this study could be detected, leading to valid measurement outcomes. A notable advantage of the LAS Grimm model 1.109 is its ability to measure PM concentrations for each tobacco product in real-time with a resolution of 6 seconds through light scattering. In contrast, reference methods employed by the US EPA and the European Committee for Standardization for PM measurement often rely on gravimetric techniques that involve 24-hour samples, but also real-time monitoring options such as the tapered element oscillating microbalance (TEOM) and the Grimm model EDM 180, which likewise uses light scattering, are available.^63,64^ The measurement results obtained using the LAS Grimm model 1.109 have been shown to be quite comparable to those from gravimetric methods, TEOM, and the Grimm model EDM 180.^65^

## Conclusion

This study reaffirmed a notable increase in PM concentrations in enclosed environments because of tobacco product smoking. In addition, this study revealed differences in PM emissions from cigarettes produced in different countries. Out of the 16 cigarette brands evaluated, five Turkish brands generated the highest levels of PM. Conversely, the five brands with the lowest PM emissions included all four German brands along with one Turkish brand, with the German brands producing less PM than their similar Turkish counterparts. While the health risks associated with PM are well-documented, tobacco companies are not required to disclose PM emissions from their products. The findings related to PM concentration in cigarettes should encourage policymakers to enhance tobacco control measures not only in Türkiye but globally. Future studies examining PM pollution from tobacco products in various countries could provide compelling evidence to strengthen tobacco control efforts, protect non-smokers, and enhance education and awareness initiatives.

## Supplemental Material


Supplemental Material - Particulate Matter Emissions of Turkish Cigarettes in Comparison to German Brands – An Aerosol Spectrometric Study
Supplemental Material for Particulate Matter Emissions of Turkish Cigarettes in Comparison to German Brands – An Aerosol Spectrometric Study by Markus Braun, Nicole Merkel, Doris Klingelhöfer, Janis Dröge and David A. Groneberg in Tobacco Use Insights.

## Data Availability

Data will be made available on request. For requesting data, please write to the corresponding author.*
