# Supplementary material for: Particulate Matter Emissions of Turkish Cigarettes in Comparison to German Brands – An Aerosol Spectrometric Study
Source: Tob Use Insights. 2026 Sep 8;19:1179173X261473110. doi: 10.1177/1179173X261473110 (PMC13554688; doi:10.1177/1179173X261473110)
Supplement: Supplementary file 1 [file sj-pdf-1-tui-10.1177_1179173X261473110.pdf]

Test for normal distribution

	RefCig	Tekel 2000 TR	Muratti TR	Monte Carlo TR	Kent TR	Viceroy TR	Lark Blue TR	Parliament TR	L&M TR	Camel TR	Marlboro TR	Davidoff Ivory TR	L&M DE	Camel DE	Marlboro DE	Davidoff DE
**D'Agostino & Pearson test**																
K2	2,225	0,2288	0,6681	3,348	4,922	6,758	2,882	8,324	3,353	0,6056	1,076	4,184	1,558	1,026	2,896	1,625
P value	0,3287	0,8919	0,7160	0,1875	0,0853	0,0341	0,2367	0,0156	0,1870	0,7387	0,5839	0,1234	0,4588	0,5988	0,2350	0,4437
Passed normality test (alpha=0.05)?	Yes	Yes	Yes	Yes	Yes	No	Yes	No	Yes	Yes	Yes	Yes	Yes	Yes	Yes	Yes
P value summary	ns	ns	ns	ns	ns	*	ns	*	ns	ns	ns	ns	ns	ns	ns	ns
**Anderson-Darling test**																
A2*	0,2982	0,1542	0,4333	0,4661	0,4975	1,180	0,3754	1,251	0,2966	0,2329	0,3721	0,5402	0,4336	0,2562	0,4908	0,4337
P value	0,5649	0,9501	0,2793	0,2293	0,1925	0,0035	0,3866	0,0023	0,5644	0,7745	0,3938	0,1493	0,2788	0,6954	0,2003	0,2786
Passed normality test (alpha=0.05)?	Yes	Yes	Yes	Yes	Yes	No	Yes	No	Yes	Yes	Yes	Yes	Yes	Yes	Yes	Yes
P value summary	ns	ns	ns	ns	ns	**	ns	**	ns	ns	ns	ns	ns	ns	ns	ns
**Shapiro-Wilk test**																
W	0,9689	0,9815	0,9525	0,9353	0,9302	0,8736	0,9594	0,8689	0,9540	0,9766	0,9598	0,9487	0,9494	0,9650	0,9470	0,9437
P value	0,5088	0,9135	0,2851	0,1421	0,0879	0,0062	0,4023	0,0041	0,3074	0,8111	0,4100	0,2348	0,2429	0,5238	0,2147	0,1799
Passed normality test (alpha=0.05)?	Yes	Yes	Yes	Yes	Yes	No	Yes	No	Yes	Yes	Yes	Yes	Yes	Yes	Yes	Yes
P value summary	ns	ns	ns	ns	ns	**	ns	**	ns	ns	ns	ns	ns	ns	ns	ns
**Kolmogorov-Smirnov test**																
KS distance	0,09477	0,07858	0,1209	0,1343	0,1592	0,2136	0,1190	0,2049	0,1026	0,1265	0,1056	0,1462	0,1198	0,1003	0,1224	0,1451
P value	>0,1000	>0,1000	>0,1000	>0,1000	>0,1000	0,0060	>0,1000	0,0082	>0,1000	>0,1000	>0,1000	>0,1000	>0,1000	>0,1000	>0,1000	>0,1000
Passed normality test (alpha=0.05)?	Yes	Yes	Yes	Yes	Yes	No	Yes	No	Yes	Yes	Yes	Yes	Yes	Yes	Yes	Yes
P value summary	ns	ns	ns	ns	ns	**	ns	**	ns	ns	ns	ns	ns	ns	ns	ns
Number of values	30	25	25	23	25	24	25	25	25	25	25	25	25	25	25	25
